# Evaluation and Characterization of Bacterial Metabolic Dynamics with a Novel Profiling Technique, Real-Time Metabolotyping

**DOI:** 10.1371/journal.pone.0004893

**Published:** 2009-03-16

**Authors:** Shinji Fukuda, Yumiko Nakanishi, Eisuke Chikayama, Hiroshi Ohno, Tsuneo Hino, Jun Kikuchi

**Affiliations:** 1 RIKEN Research Center for Allergy and Immunology, Suehiro-cho, Yokohama, Japan; 2 International Graduate School of Arts and Sciences, Yokohama City University, Suehiro-cho, Yokohama, Japan; 3 RIKEN Plant Science Center, Suehiro-cho, Yokohama, Japan; 4 Department of Life Science, Meiji University, Tama-ku, Kawasaki, Japan; 5 Graduate School of Bioagricultural Sciences, Nagoya University, Furo-cho, Nagoya, Japan; RIKEN Genomic Sciences Center, Japan

## Abstract

**Background:**

Environmental processes in ecosystems are dynamically altered by several metabolic responses in microorganisms, including intracellular sensing and pumping, battle for survival, and supply of or competition for nutrients. Notably, intestinal bacteria maintain homeostatic balance in mammals via multiple dynamic biochemical reactions to produce several metabolites from undigested food, and those metabolites exert various effects on mammalian cells in a time-dependent manner. We have established a method for the analysis of bacterial metabolic dynamics in real time and used it in combination with statistical NMR procedures.

**Methodology/Principal Findings:**

We developed a novel method called real-time metabolotyping (RT-MT), which performs sequential ^1^H-NMR profiling and two-dimensional (2D) ^1^H, ^13^C-HSQC (heteronuclear single quantum coherence) profiling during bacterial growth in an NMR tube. The profiles were evaluated with such statistical methods as Z-score analysis, principal components analysis, and time series of statistical TOtal Correlation SpectroScopY (TOCSY). In addition, using 2D ^1^H, ^13^C-HSQC with the stable isotope labeling technique, we observed the metabolic kinetics of specific biochemical reactions based on time-dependent 2D kinetic profiles. Using these methods, we clarified the pathway for linolenic acid hydrogenation by a gastrointestinal bacterium, *Butyrivibrio fibrisolvens*. We identified *trans*11, *cis*13 conjugated linoleic acid as the intermediate of linolenic acid hydrogenation by *B. fibrisolvens*, based on the results of ^13^C-labeling RT-MT experiments. In addition, we showed that the biohydrogenation of polyunsaturated fatty acids serves as a defense mechanism against their toxic effects.

**Conclusions:**

RT-MT is useful for the characterization of beneficial bacterium that shows potential for use as probiotic by producing bioactive compounds.

## Introduction

A huge number of microorganisms are known to colonize and form complex microbial ecosystems within the human and animal gut [1]–[5]. It is generally accepted that microbial ecosystems associated with humans or animals have a direct influence on the host's health. Gut microbiota possess a number of metabolic capabilities that are lacking in the host and thus, can be viewed as indispensable to the maintenance of health. Gut microbes contribute to host nutrition by producing organic acids from undigested carbohydrates and by synthesizing bioactive substances, including vitamins [6], [7]. Intestinal bacteria and their metabolites, including short-chain fatty acids (SCFAs), butyric acid in particular, exert significant physiological effects on the host by controlling the differentiation and proliferation of intestinal epithelial cells, providing energy to epithelial cells, modulating the immune system, and protecting against pathogens [6]–[10]. An imbalance of intestinal microbiota can predispose individuals to a variety of disease states ranging from inflammatory bowel disease to allergy and obesity [3], [4], [11].

Recently, many probiotics have emerged and their effects on human health have been partially demonstrated [12]–[15]. As a single probiotic bacterium can exert several effects on human health mainly through the improvement of host innate immune responses by producing several bioactive substances[16]–[21], an understanding of the metabolic dynamics of purified bacterium is absolutely essential. However, the mechanisms underlying their effects have not been well elucidated and thus, the functional analysis of probiotics is eagerly anticipated. Because metabolites that can be utilized similarly by host cells and bacterial cells are considered to be one of the most important factors to help us understand the effectiveness of probiotics [22], [23], we need to establish a method for the analysis of metabolic dynamics.

Since bacteria grow in the gut, it is desirable to analyze their metabolic dynamics at the growth phase to elucidate their ability to produce organic acids and physiologically active substances. The multiple high-throughput metabolic analysis of *Escherichia coli* K12 knockdown system has been reported [24]; however, it requires bacterial whole genomic information and knockdown systems, and is not easily applicable to the estimation of bacterial metabolic dynamics.

To use bacteria as probiotics, it is necessary to screen their characteristics, such as pH resistance and the ability to produce bioactive substances [25], [26]. As screening methods are diverse and usually complicated, an appropriate strategy is desired. By characterizing bacteria based on their metabolic profiles, we would be able to know and estimate their ability to produce bioactive substances or their metabolic dynamics in response to environmental factors, such as nutrients and chemicals. Accordingly, the development of a universal bacterial evaluation system is highly awaited in food science technology.

To understand bacterial metabolic dynamics, several analytical strategies having notable technological features have been introduced [27], [28]. In addition, an important concept that is based on the global analysis of environmental metabolites, the so-called metabolic phenotype, has been reported [29]–[33]. However, these strategies are insufficient because they can observe only metabolites at a certain growth stage of living cells or under certain environmental conditions. In other words, those strategies provide only a “static” view of metabolic aspects. Most enzyme reactions are completed within a few seconds, while metabolic reactions occur on a time scale of minutes to hours and changes in microbial growth occur on a time scale of hours to days. Several techniques for monitoring living cells have been reported, including *in situ*, *ex vivo*, and *in vivo* NMR [34]–[43]. However, efficient approaches that combine such *real*-time dependent (*in vivo*) events and *statistical* approaches have not been investigated.

We have reported that a gastrointestinal bacterium, *Butyrivibrio fibrisolvens*, produces conjugated linoleic acid (CLA) and conjugated linolenic acid (CLNA) from linoleic acid (LA) and alpha-linolenic acid (LNA), respectively [44]–[47]. CLA and CLNA produced by *B. fibrisolvens* are known as health-promoting substances because of their beneficial effects on human health [48]–[53]. However, the metabolic dynamics of *B. fibrisolvens*, including CLA and CLNA production, is not well understood. Here we developed a novel profiling technique called real-time metabolotyping (RT-MT) to understand and evaluate metabolic dynamics when used in combination with real-time and statistical NMR analysis methods. As a result of performing ^13^C-labeling RT-MT experiments, we clarified the LNA hydrogenation pathway of *B. fibrisolvens* based on our accumulated knowledge in NMR analyses and stable isotope labeling techniques [54]–[58].

## Results and Discussion

### Development of RT-MT to understand bacterial metabolic dynamics and possible applications

**Figure 1:**
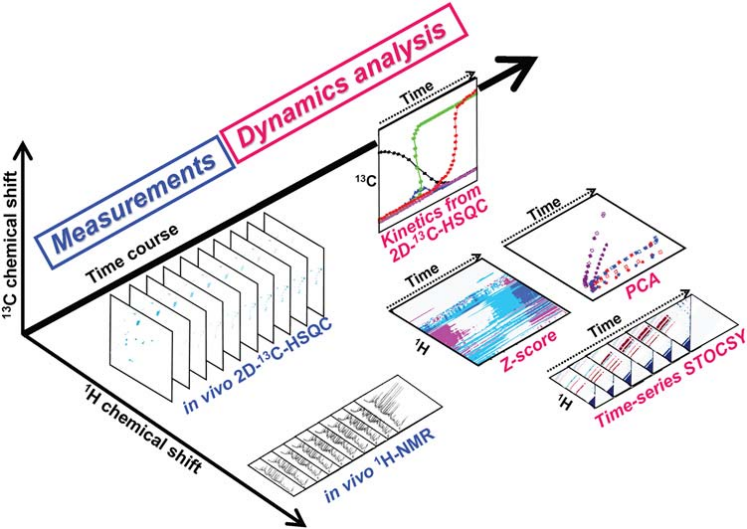
Development of real-time metabolotyping (RT-MT) to analyze bacterial metabolic dynamics. *In vivo* ^1^H-NMR (right) and two-dimensional ^1^H, ^13^C-HSQC with ^13^C labeled substrate technique (2D-^13^C-HSQC) (left) were continuously conducted during bacterial growth in an NMR tube at 37°C, and the profiling data were processed and analyzed by several statistical methods, such as Z-score analysis, principal components analysis (PCA), and time series of statistical total correlation spectroscopy (STOCSY). From the results of statistical analyses of *In vivo* ^1^H-NMR profiling data, we extracted meaningful data related to time-varying information. Furthermore, from the results of *in vivo* 2D-^13^C-HSQC profiling data, we revealed the metabolic kinetics of specific metabolic reactions.

We have developed RT-MT to understand and evaluate the metabolic dynamics of several bacterial strains (Fig. 1). The most important point of this method is that the time-dependent metabolic profiles from ^1^H-NMR and ^1^H, ^13^C-HSQC sequential observations during bacterial growth in an NMR tube are calculated and evaluated with several statistical methods, such as Z-score analysis, principal components analysis (PCA), and statistical total correlated spectroscopy (STOCSY). It is considered that the statistical analyses of time-dependent metabolic profiles would show meaningful biological data related to metabolic dynamics. In addition, time-dependent two-dimensional ^1^H, ^13^C-HSQC with ^13^C labeled substrates (2D-^13^C-HSQC) profiles could be used to generate three-dimensional metabolic kinetic profiles. Time-dependent 2D-^13^C-HSQC can provide very detailed information of specific metabolic dynamics. As anaerobic conditions can be easily created in NMR tubes, RT-MT can be applied to the analysis of anaerobic environments, such as soil, industrial plant, and animal gut. RT-MT is expected to improve our understanding of metabolic dynamics that would be necessary for the extraction of particular characteristics of metabolic changes.

### Use of time-dependent ^1^H-NMR RT-MT to assess bacterial characteristics

#### a) Z-score analysis of *B. fibrisolvens* strains

**Figure 2:**
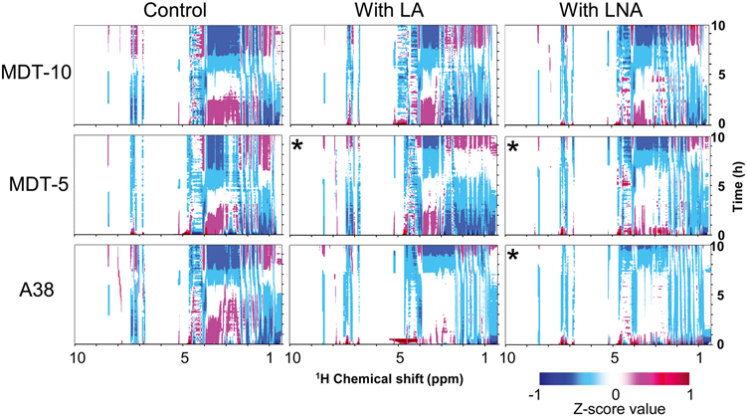
Z-score analysis of time-dependent ^1^H-NMR profiles of three *B. fibrisolvens* strains incubated with or without LA or LNA. A total of 120 continuously acquired *in vivo* ^1^H-NMR spectra were used in each Z-score analysis. MDT-10, MDT-5, and A38 were grown in anaerobic culture (control), with 2.5 mM LA, or with 2.5 mM LNA. Asterisks mean low concentration (0.6 mM) of FAs, because MDT-5 did not grow in the presence of 2.5 mM LA or 2.5 mM LNA, and A38 did not grow in the presence of 2.5 mM LNA. Data are representative of three independent experiments. Contour levels (Z-score values) defined by different colors are indicated at the bottom.

Z-score analysis of three *B. fibrisolvens* strains revealed their metabolic dynamics, including time-varying factors (Fig. 2). All samples showed remarkable changes of the chemical shifts in the 1 to 4 ppm region, which were derived from lipids, organic acids, and sugars, and around 8 ppm, which were derived from formic acid. In particular, signals around 2 ppm, which were attributed mainly to acetic acid present in abundance, were slightly shifted downfield with decreasing culture pH. A negative correlation was observed between glucose concentration, which was calculated from the ^1^H-NMR standard curve (data not shown), and bacterial growth rate (SI Fig. S1). It was possible to calculate the transition to acidic culture pH due to organic acid production by bacteria from the chemical shift changes of bacterial metabolites containing a carboxylic group (SI Fig. S2). Furthermore, Z-score analysis clearly showed changes in the chemical shifts of metabolites present in small amounts, which appeared as unidentified signals in the low field region (8.3–8.6 ppm) during bacterial growth.

Interestingly, the presence or absence of linoleic acid (LA) or linolenic acid (LNA) contributed more significantly to the difference in Z-score profiles than the difference in strain under the same conditions. Anaerobic *B. fibrisolvens* strains exhibit different metabolic activities of and tolerance activities to exogenous lipids related to CLA production [47], and the remarkable differences of the Z-score profiles among control, LA, and LNA conditions are a reflection of the difference in metabolic activities of the strains. From these findings, we speculated that RT-MT may be applicable to chemical biological research [59], [60] as well as to silent phenotype analysis [61] to screen for objective chemicals and phenotypes, because RT-MT can easily monitor metabolic changes based on specific reactions and responses by the addition of several compounds.

In cultures with LA or LNA, Z-score profiles mainly around the 1 to 4 ppm region were changed compared to the cultures without LA or LNA (Fig. 2). In particular, Z-score variations around 3.5 ppm represent variations in the amounts of sugars. The results show that the relative sugar consumption rates of MDT-5 and A38 strains grown in the presence of LNA, as well as of A38 grown in the presence of LA, were lower than those of control cultures. This result suggests that fatty acids (FAs) inhibit bacterial growth, as has been reported previously [62]. However, the growth of MDT-10 was not markedly inhibited by LA or LNA. Our past data showed that MDT-10 can rapidly metabolize LA and LNA to vaccenic acid (VA), which is less toxic than LA and LNA to bacteria [45]. Therefore, MDT-10 is considered to be resistant to FAs. From these results, we consider that RT-MT is useful for the rough visualization of bacterial metabolic dynamics in response to exogenous effectors.

#### b) PCA of *B. fibrisolvens* strains

**Figure 3:**
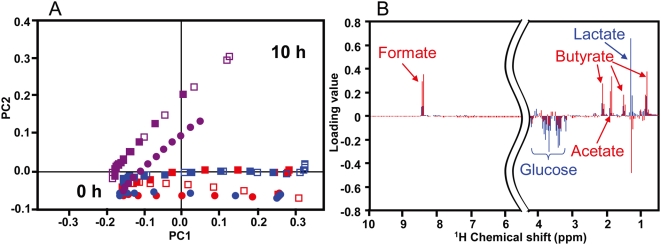
Principal components analysis (A) and loading plot analysis (B) of three strains of *B. fibrisolvens*. MDT-10 (red), MDT-5 (blue), and A38 (purple) are shown. Data under conditions of LA addition (open squares), LNA addition (close squares), or no addition (close circles) were calculated. Data are representative of three independent experiments. (A) Hourly ^1^H-NMR profiling data of three strains of *B. fibrisolvens* incubated with or without LA or LNA were analyzed. Contributions of PC1 and PC2 were 77.0% and 11.6%, respectively. All 0 h samples were assembled at the left side of Figure 3A, indicating that metabolic conditions before bacterial growth were similar. However, after 10 h, the samples were located in the right side of Figure 3A, suggesting that PC1 means bacterial growth and PC2 means metabolic differences among *B. fibrisolvens* strains. (B) Blue peaks and words indicate PC1 contribution and those in red indicate PC2 contribution. The 4.2–5.2 ppm region was omitted to eliminate the effects of imperfect water suppression.

Different from Z-score analysis, it is possible to detect characteristic metabolites by PCA. Interestingly, the differences in PCA profiles were more remarkable among the different strains than among the different medium conditions (Fig. 3-A). The PCA profiles of the three strains revealed that the metabolic characteristics of MDT-10 and MDT-5 were similar, while those of A38 were quite different. We calculated the first two components that contained principal components 1 and 2 contributing to 77.0% and 11.6% of the data variance, respectively. The strains were well separated primarily in PC2 and appeared to form two groups of A38 and of MDT-10 and MDT-5. In contrast, PC1 was dominated by the time course, which is common to the three strains. The spectral regions that contributed most to the difference between the two groups are shown in the corresponding loading plot (Fig. 3-B). Metabolites that exerted the greatest influence on this separation were lactate and glucose in PC1 and butyrate, acetate, and formate in PC2 (Fig. 3-B). As the three strains grew, they consumed glucose and produced lactate; however, butyrate, acetate, and formate production was different between the two groups. It is well known that *B. fibrisolvens* strains could be classified into Type I (high butyrate and formate production) or Type II (high lactate production) based on their fermentation products but not their 16S rDNA sequences [63]–[65]. It has been reported that these differences may be the basis for genetic differences related to the butyrate production pathway [65]. Therefore, this analytical method could be used to characterize bacterial fermentation patterns derived from functional genetic information.

#### c) STOCSY analysis of *B. fibrisolvens* MDT-10

In order to clarify the pathway of LNA metabolism by *B. fibrisolvens* MDT-10, we next analyzed the time series of STOCSY spectra [66], and the results showed time-dependent metabolic changes in bacterial growth at hourly intervals (SI Fig. S3). For the spectra obtained without LNA, a negative correlation was observed between glucose (around 3.5 ppm) and organic acids (around 1.5 ppm) in the spectra at 3 to 6 h; the correlation corresponded to the activity during the growth period of MDT-10, because the growth reached a maximum after 6 h (SI Fig. S1). Moreover, the negative correlation vanished after 8 h in the spectra of cultures grown without LNA, whereas the negative correlation in the spectra of cultures grown with LNA existed until 9 h in spite of the addition of LNA, which has a stronger growth inhibitory effect than LA. Negative correlations in the spectra of the cultures grown with LNA were low at 3 to 9 h compared to control values (grown without LNA), suggesting that the growth of MDT-10 was inhibited by LNA, and that MDT-10 grew after LNA was metabolized to VA, which is much less cytotoxic than LNA [47].

**Figure 4:**
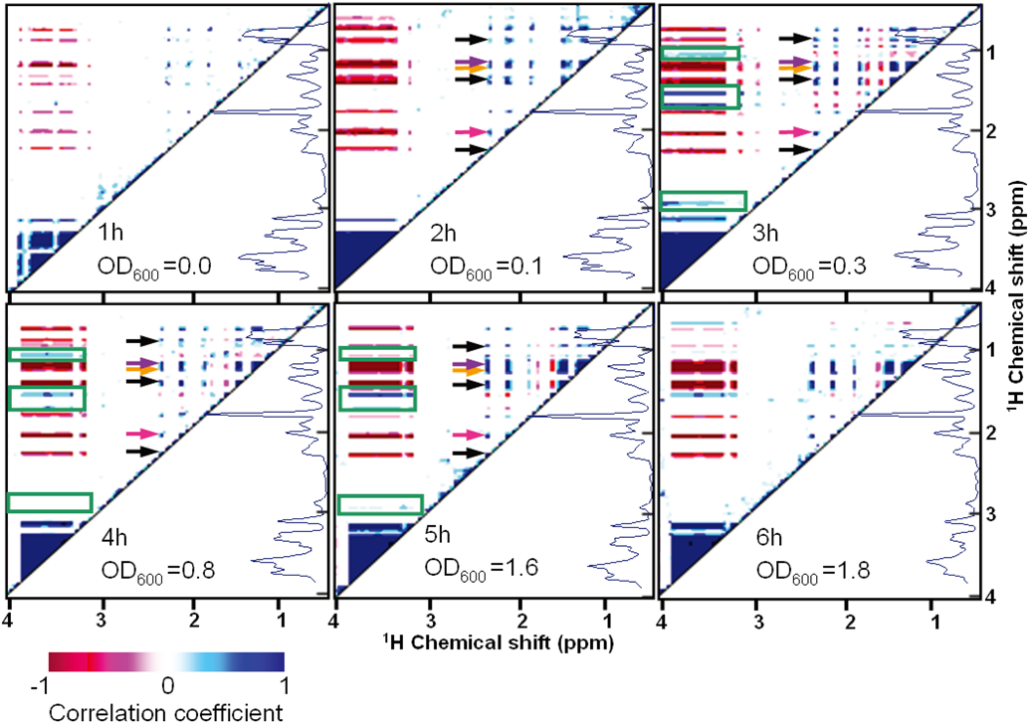
Time series of STOCSY analysis of *B. fibrisolvens* MDT-10 incubated with LNA. During these one-hour experiments, a total of twelve continuously acquired *in vivo* ^1^H-NMR spectra were used for STOCSY analysis. Spectra appearing on the right side are the sum of the twelve *in vivo* ^1^H-NMR spectra measured in every one-hour experiment. Contour levels (STOCSY correlations) defined by different colors are indicated at the bottom. Blue represents high positive correlation and red represents low. Optical density (OD) is a measure of bacterial growth. Arrows indicate butyrate (black), lactate (orange), acetate (pink), and ethanol (purple). Signals surrounded by green squares are assumed to be derived from LNA or its metabolites. Data are representative of three independent experiments.

Analyzing the metabolic response of MDT-10 to LNA, signals in the 1 to 3 ppm region were greatly changed and some of the compounds were successfully identified. For example, several organic acids produced from glucose were identified, and some signals, such as those at 1.2, 1.5, 1.8, and nearby 2.8 ppm, showed a positive correlation with glucose consumption (Fig. 4). As those signals were assumed to be derived from LNA or its metabolites, we next performed 2D ^1^H, ^13^C-HSQC analysis using U-^13^C_18_ LA and U-^13^C_18_ LNA.

### Utilization of time-dependent 2D-^13^C-HSQC RT-MT to narrow down biochemical reactions analyzed

To monitor the specific metabolic dynamics, we developed time-dependent 2D- ^13^C-HSQC with U-^13^C_18_ LA and U-^13^C_18_ LNA. As the time-dependent 2D-^13^C-HSQC profiles could be used to generate three-dimensional metabolic profiles, it is possible to understand very detailed information of specific metabolic kinetics. It is well known that NMR signal intensities can be altered by such factors as the mobility of molecules, pH, and sample homogeneity [67]–[73]. However, our results indicated that the normalized signal intensities of ^13^C-labeled substrate were little affected by bacterial optical density, differences in NMR data processing, and culture pH (SI Tables S1 and S2). In addition, the variability of triplicate experiments was caused by differences in the volume of pre-cultured bacterial medium that was initially inoculated into the NMR tube. NMR tuning and shimming could also change NMR signal intensities and line widths; however, we found that these were constant during *in vivo* NMR observation. Therefore, the results of our kinetic analysis were highly reliable.

In the case of RT-MT with U-^13^C_18_ LA and U-^13^C_18_ LNA, we state two reasons why the signal intensities were largely unaltered. First, the culture media contained large amounts of molecules, including sugars, peptides, organic acids, minerals, and vitamins, for bacterial growth [21]. Second, to dissolve U-^13^C_18_ LA and U-^13^C_18_ LNA in culture media, they were mixed and adsorbed to bovine serum albumin (BSA) [47]. It is for these two reasons that we conclude that the signal intensities of U-^13^C_18_ LA and U-^13^C_18_ LNA were largely unaltered.

#### a) Dynamics of LA metabolism by *B. fibrisolvens* MDT-10

It is well known that *B. fibrisolvens* hydrogenates LA and LNA to VA in order to decrease their cytotoxicity [62]. Our past findings also suggest that LA hydrogenation activity and tolerance ability to LA are correlated [47]. When we monitored LA metabolic kinetics of MDT-10 using time-dependent 2D-^13^C-HSQC RT-MT, we found that the growth rate of MDT-10 was low at the initial stage of growth when LA concentration was high. However, the growth rate increased gradually as LA was reduced to VA through the transient accumulation of *cis*9, *trans*11-CLA (SI Fig. S4). As mentioned above, LA or CLA hydrogenation to VA may be a defense response. These results are consistent with our past data; therefore, time-dependent 2D-^13^C-HSQC RT-MT is useful for understanding metabolic kinetics.

#### b) Dynamics of LNA metabolism by *B. fibrisolvens* MDT-10

**Figure 5:**
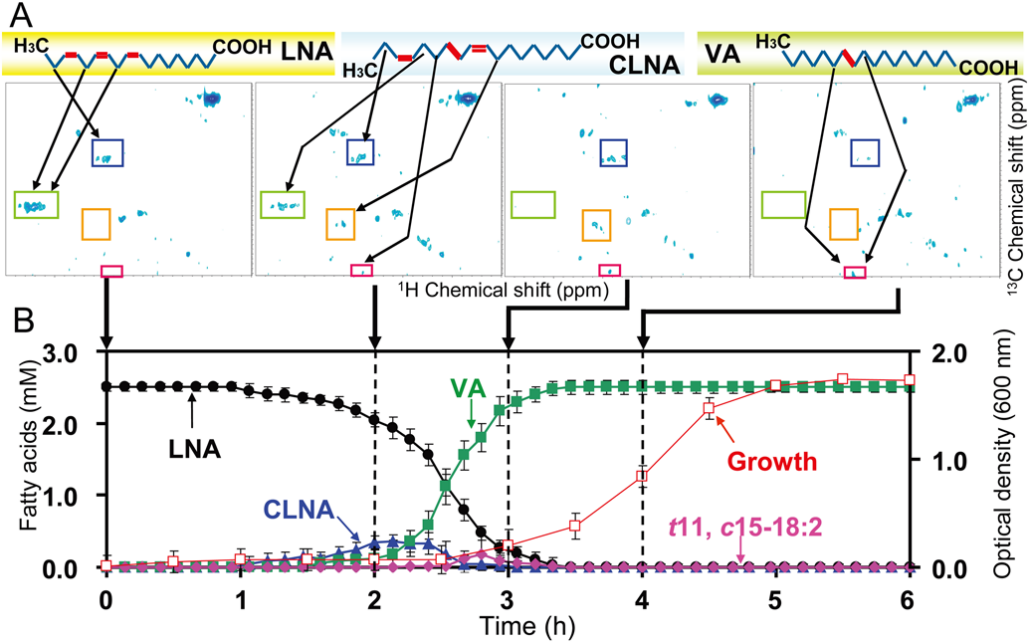
LNA metabolic dynamics of *B. fibrisolvens* MDT-10 analyzed by time-dependent 2D-^13^C-HSQC RT-MT. The NMR tube was anaerobically inoculated with *B. fibrisolvens* MDT-10 and U-^13^C_18_ LNA (2.5 mM) was added to follow LNA hydrogenation reactions in *in vivo* 2D-^13^C-HSQC RT-MT. A: Typical 2D HSQC spectra (sequential growth at 0, 2, 3, and 4 hours) are shown. 2D HSQC spectra were observed every 8 minutes. Arrows in upper panel indicate C-H structures corresponding to the signals. Signal intensities were calculated based on their standard curves. Arrows pointing to lower panel (B) indicate 2D HSQC spectra measured at the indicated time point. B: LNA metabolic dynamics of *B. fibrisolvens* MDT-10 calculated from time-dependent 2D-^13^C-HSQC RT-MT. LNA (black circles), CLNA (blue triangles), *t*11, *c*15–18:2 (pink diamonds), VA (green squares), and bacterial growth (open red squares) are shown. Mean values of triplicate experiments are shown.

**Figure 6:**
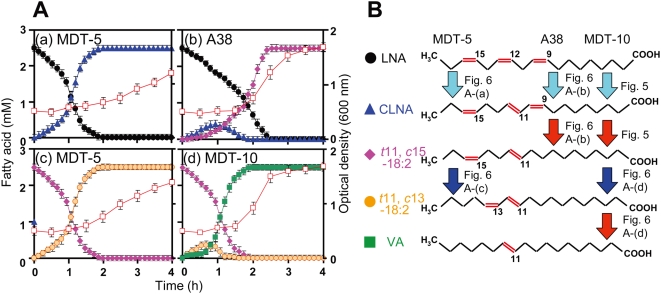
Metabolic dynamics of LNA and *t*11, *c*15–18:2 in three strains of *B. fibrisolvens* analyzed by time-dependent 2D-^13^C-HSQC RT-MT. A: When the initial OD was approximately 0.5, U-^13^C_18_ LNA or *t*11, *c*15–18:2 (2.5 mM) was added to cultures to avoid growth inhibition. LNA (black circles), CLNA (blue triangles), *t*11, *c*15–18:2 (pink diamonds), *t*11, *c*13–18:2 (orange circles), VA (green squares), and bacterial growth (open red squares) are shown. A38 did not metabolize *t*11, *c*15–18:2 (data not shown). Mean values of triplicate experiments are shown. B: LNA hydrogenation pathway of three strains of *B. fibrisolvens* deduced from the results of this study. Chemical structures and location of double bonds of LNA and its metabolites are shown. Arrows indicate enzymes responsible for each reaction. Light blue, red, and dark blue arrows represent LNA isomerase, CLA-R, and *t*11, *c*15–18:2 isomerase, respectively.

Our past work has demonstrated that the bacterial growth inhibitory activity of unsaturated FAs increases with increasing degree of unsaturation [47]. Therefore, we next analyzed the metabolic dynamics of LNA, which is more unsaturated than LA, using MDT-10. Similar to the case of LA (SI Fig. S4), MDT-10 growth was suppressed at the initial growth stage when LNA concentration was high, but was gradually improved as LNA was hydrogenated to VA (Fig. 5). The intermediates of LNA hydrogenation were identified as CLNA, *trans*11, *cis*15–18:2 (*t*11, *c*15–18:2), and VA. When similar analysis was performed with MDT-5 and A38, the final product of LNA hydrogenation was CLNA and *t*11, *c*15–18:2, respectively (Fig. 6-A). Previously, we reported that strain TH1 produced CLNA and *t*11, *c*15–18:2 as the intermediates of LNA hydrogenation [46], and we also observed that strains ATCC19171 and ATCC51255 produced CLNA and *t*11, *c*15–18:2 as intermediates (data not shown). Thus, the LNA hydrogenation pathway may be similar among *B. fibrisolvens* strains except A38 and MDT-5.

### Application of time-dependent 2D-^13^C-HSQC RT-MT to protein functional analysis based on biochemical reactions: LNA hydrogenation pathway in *B. fibrisolvens* MDT-10

We found previously that CLA-R from MDT-10 reduces CLNA to *t*11, *c*15–18:2 but did not reduce *t*11, *c*15–18:2 to VA [44]. Therefore, we surmised that *t*11, *c*15–18:2 is isomerized once to another form, and then reduced to VA. When MDT-5, which has no CLA-R activity, was grown with *t*11, *c*15–18:2 that was produced from U-^13^C_18_ LNA by A38, only an unidentified 18:2 FA was produced (Fig. 6-A), and A38 did not metabolize *t*11, *c*15–18:2 further (data not shown). Meanwhile, MDT-10 converted the unidentified 18:2 FA produced by MDT-5 into VA. These results are consistent with the information for LNA metabolism described above.

The unidentified 18:2 FA produced by MDT-5 was shown to be an isomer of *t*11, *c*15–18:2, as examined by gas chromatography-mass spectrometry (GC-17A, Shimadzu Co., Kyoto, Japan) (data not shown). This FA exhibited absorbance at 233 nm in reversed-phase HPLC (Prominence, Shimadzu Co., Kyoto, Japan) (data not shown), indicating that the FA has conjugated double bonds. When we compared the structures of the intermediates produced by U-^13^C_18_ LNA hydrogenation by 2D ^1^H, ^13^C-HSQC profiling, the unidentified 18:2 FA was found to have *trans* and *cis* double bonds (SI Fig. S5). In addition, since purified CLA-R from MDT-10 reduced this FA directly to VA, which has a *trans*-11 double bond, this FA would have at least one *trans*-11 double bond (data not shown). TOCSY analysis revealed that CH_3_ and CH of the double bond of the unidentified 18:2 FA were correlated, whereas CH_3_ and CH of the double bond of VA were not correlated, suggesting that the double bonds of this FA are located at positions 11 and 13, respectively (SI Fig. S6). Thus, 18:2 FA was identified as *t*11, *c*13-CLA. It has been suggested that conjugated 18:2 isomers in general have health-promoting activities [48]. *t*11, *c*13-CLA was found in milk of cows grazing mountain pasture, which is rich in LNA [74]. Taken together, we conclude that the LNA hydrogenation pathway in the three *B. fibrisolvens* strains is as shown in Fig. 6-B. It is noteworthy that the CLA-R from MDT-10 hydrogenates both CLNA and *t*11, *c*13-CLA.

It has been reported that LA isomerase (LA-I) purified from A38 isomerized not only LA to CLA but also LNA to CLNA [75]–[77]. As mentioned above, our experiments with A38 showed that the final product of LNA hydrogenation is *t*11, *c*15–18:2, indicating that LA-I of A38 is unable to isomerize *t*11, *c*15–18:2 to other isomers, such as *t*11, *c*13–18:2. Although we have not purified LA-I from MDT-10 or MDT-5, their LA-I may be similarly unable to isomerize *t*11, *c*15–18:2. Thus, we speculate that MDT-10 and MDT-5 have a specific *t*11, *c*15–18:2 isomerase that differs from LA-I.

### Possible applications to applied sciences

We demonstrated that metabolic changes of the three strains of *B. fibrisolvens* in response to unsaturated FAs can be evaluated by RT-MT. Therefore, this method is a powerful tool for the investigation and estimation of bacterial metabolic dynamics. By monitoring bacterial metabolic dynamics in real time, we would be able to understand their metabolic activities and evaluate their effectiveness as candidates for probiotics. The characteristics of the three *B. fibrisolvens* strains are as follows: MDT-5 has higher CLNA-producing ability than the other two strains (Fig. 6-A), but its growth is inhibited at high LA and LNA concentrations (Fig. 2). A38 produces a large amount of butyrate having several physiological activities [21], compared to the other two strains (Fig. 3). MDT-10 metabolizes LA and LNA to VA and produces *t*11, *c*13 conjugated FA from LNA (Figs. 2, 6, and SI Fig. S4). We previously proposed that MDT-5 and MDT-10 may be beneficial as probiotics for animals including humans, by acting to increase CLA and CLNA production in the intestine [45], [46], and that MDT-10 may also be useful to augment butyrate production in the intestine [21]. Thus, to improve bacterial production of bioactive substances, a solid understanding of metabolic dynamics is important.

RT-MT can be used to understand metabolic pathways and may have applications in industry as an innovative bacterial monitoring system. Bacterial fermentation products have been examined for possible use as renewable energy and materials in the biorefinery industry [78] and therefore, RT-MT might be extended to strain screening, strain improvement, and evaluation for metabolic engineering [36], [79]–[81]. Further applications include process monitoring in the food industry and environmental management, including wine fermentation [82], lactobacillus fermentation [82], [83], and bioremediation [28]. It is considered that the detection limit of metabolites by NMR is generally much lower than that of other apparatuses, such as GC-MS and LC-MS. However, the amounts of metabolites affecting human health, such as SCFAs produced by colonized gut microbes, are considerably high [84]. If only the quantification of metabolites could be accomplished, we would be able to partially predict metabolic pathways and use them in combination with genomic information. Thus, *in vivo* NMR techniques are considered to be useful for the characterization of bacterial strains, and our newly proposed method, RT-MT, may open a new avenue for basic life science, industry, and environmental management.

## Materials and Methods

### Bacterial strains, reagents, and culture conditions

Sources of *B. fibrisolvens* strains (A38, MDT-5, and MDT-10) were described previously [45]–[47]. Unless otherwise stated, each strain was grown in 30 mL serum vials containing growth medium (15 mL) with 5 g/L glucose. Details of culture medium, growth conditions, and procedures were as described previously [21], [85]. In the case of *in vivo* NMR measurement, the strains were cultured in a 5 mmф NMR tube containing 1 mL of growth medium, 10% (v/v) D_2_O, and 1 mM sodium 2,2- dimethyl-2- silapentane-5-sulfonate (DSS).

U-^13^C_18_ LA and LNA, D_2_O, and DSS were purchased from Shoko Tsusho Ltd. (Tokyo, Japan). FAs were added as a mixture with BSA, which was prepared as described previously [47].

### 1D and 2D NMR measurements

All NMR spectra were recorded on a Bruker DRX-500 spectrometer operating at 500.03 MHz ^1^H frequency with the temperature of NMR samples maintained at 310 K. For 1D *in vivo* ^1^H-NMR, spectra were observed every 5 minutes and residual water signals were suppressed by Watergate pulse sequence with 1.2 second repetitive time. 2D ^13^C-HSQC spectra were measured every 8 minutes according to the method of Kikuchi and Hirayama [86]. Briefly, 1D NMR spectra were measured on a Bruker DRX-500 spectrometer equipped with a ^1^H inverse probe with triple axis gradient. All 2D ^13^C-HSQC spectra were recorded on a Bruker DRX-500 spectrometer equipped with a ^1^H inverse cryogenic probe with Z-axis gradient. A total of 32 complex f1 (^13^C) and 1024 complex f2 (^1^H) points were recorded with 8 scans per f1 increment. Spectral widths were 2,640 Hz and 5000 Hz for f1 and f2, respectively. To quantify signal intensities, a Lorentzian-to-Gaussian window with a Lorentzian line width of 10 Hz and a Gaussian line width of 15 Hz was applied in both dimensions, prior to Fourier transformation. A fifth-order polynomial baseline correction was subsequently applied in f1 dimension. The indirect dimension was zero-filled to 512 points in the final data matrix. NMR spectra were processed using NMRPipe software [87], [88].

### Quantitative statistical analysis of 1D ^1^H-NMR spectra

1D ^1^H-NMR data were reduced by subdividing spectra into sequential 0.04 ppm designated regions between ^1^H chemical shifts of 0.5 to 10.0. After exclusion of water resonance, each region was integrated and normalized to the total of all resonance integral regions. 2D spectral assignments were performed using customized software (Chikayama and Kikuchi, unpublished data).

The Z-score matrix that was used to visualize sequential changes of chemical shifts is defined as the matrix that has each element of
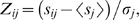
where *s_ij_* is the intensity of the *j*-th bin in the *i*-th 1D spectrum, 〈s*_j_*〉 is the average of all the intensities of the *j*-th bins in all the spectra, and *σ_j_* is the standard deviation of them. Any elements that had intensity less than 3000 (arbitrary unit) in the final Z-score matrices were replaced with zero.

PCA was run on R software. Data were visualized in the form of PC score plots and loading plots. Each coordinate on the score plot represents an individual sample and each coordinate on the loading plot represents one NMR spectral data point related to metabolites. Thus, the loading plots provide information on spectral regions responsible for the positions of coordinates or clusters of samples in the corresponding score plots.

STOCSY was also analyzed using Excel software, as described by Cloarec et al. [66]. Briefly, a STOCSY spectrum was calculated as a symmetric matrix in which an element at position (*i*, *j*) is defined as a correlation coefficient between *i*-th and *j*-th bins in a set of 1D spectra (twelve spectra each acquired on an hourly basis). A positively (negatively) higher coefficient means the existence of a positive (negative) correlation between *i*-th and *j*-th peaks throughout the spectra.

### Quantification and identification of FAs

Lipids were extracted by shaking cultures with isopropanol-isooctane-6*N* H_2_SO_4_ (20∶10∶1), as reported previously [85]. Then, the lipids were transmethylated with 5% HCl in methanol at 60°C for 20 min under N_2_ gas [85]. Methylated FAs were analyzed by gas chromatography-mass spectrometry (GC-17A, Shimadzu Co., Kyoto, Japan) as described previously [85]. In order to identify the FAs derived from LNA hydrogenation, FAs extracted from cultures were separated and collected by reversed-phase HPLC (Prominence, Shimadzu Co., Kyoto, Japan) using COSMOSIL 5C_18_-AR-II (4.6 mm I.D.×150 mm, Nacalai Tesque Inc., Kyoto, Japan).

## Supporting Information

Figure S1 Glucose concentration and growth rate of three strains of *B. fibrisolvens* incubated in NMR tubes. Glucose concentration (circles) was calculated from the signal intensities observed every 5 minutes. OD (squares) was determined hourly. Mean values of triplicate experiments are shown.
(0.42 MB TIF)

Figure S2 Dependence of pH on chemical shift mobility of acetate (A) and succinate (B). Mean values of triplicate experiments are shown.
(0.16 MB TIF)

Figure S3 Time series of STOCSY analysis of *B. fibrisolvens* MDT-10 incubated with or without LNA. During these one-hour experiments, a total of twelve continuously acquired *in vivo* ^1^H-NMR spectra were used for STOCSY analysis. Contour levels (STOCSY correlations) defined by different colors are indicated at the bottom. Blue represents high positive correlation and red represents low. Data are representative of three independent experiments.
(3.06 MB TIF)

Figure S4 LA metabolic dynamics of *B. fibrisolvens* MDT-10 analyzed by time-dependent 2D-^13^C-HSQC RT-MT. *B. fibrisolvens* MDT-10 was anaerobically inoculated in an NMR tube and U-^13^C_18_ LA (2.5 mM) was added to monitor LA hydrogenation by *in vivo* 2D-^13^C-HSQC. Mean values of triplicate experiments are shown. LA (circles), CLA (triangles), VA (squares), and bacterial growth (open squares) are shown.
(0.39 MB TIF)

Figure S5 Comparison of 2D-^13^C-HSQC spectra obtained from LNA and its metabolites. Distinct signals are shown by arrows indicating Δ^15^ double bond (red), *cis* double bond (purple), and *trans* double bond (green). Unidentified 18:2 has *trans* and *cis* double bonds, but no Δ^15^ double bond.
(0.37 MB TIF)

Figure S6 Comparison of TOCSY spectra of LNA metabolites. A: CLNA (*c*9, *t*11,*c*15–18:3), B: *t*11,*c*15–18:2, C: VA (*t*11–18:2), D: Unidentified 18:2. Black line indicates correlation between CH_3_ (brown square) and CH (green square). It was shown that unidentified 18:2 exhibits correlation between CH_3_ and CH, meaning that one of the double bonds of this FA is located more closely to the methyl group side than Δ^11^.
(1.37 MB TIF)

Table S1 Fluctuation of signal intensities of methylene group of U-^13^C_18_ LA under various *in vivo* NMR conditions.
(0.02 MB DOC)

Table S2 Fluctuation of signal intensities of methylene group of U-^13^C_18_ LA under various pH conditions.
(0.02 MB DOC)
