# Supplementary figures and images for: Evaluation and Characterization of Bacterial Metabolic Dynamics with a Novel Profiling Technique, Real-Time Metabolotyping
Source: PLoS One. 2009 Mar 16;4(3):e4893. doi: 10.1371/journal.pone.0004893 (PMC2654759; doi:10.1371/journal.pone.0004893)

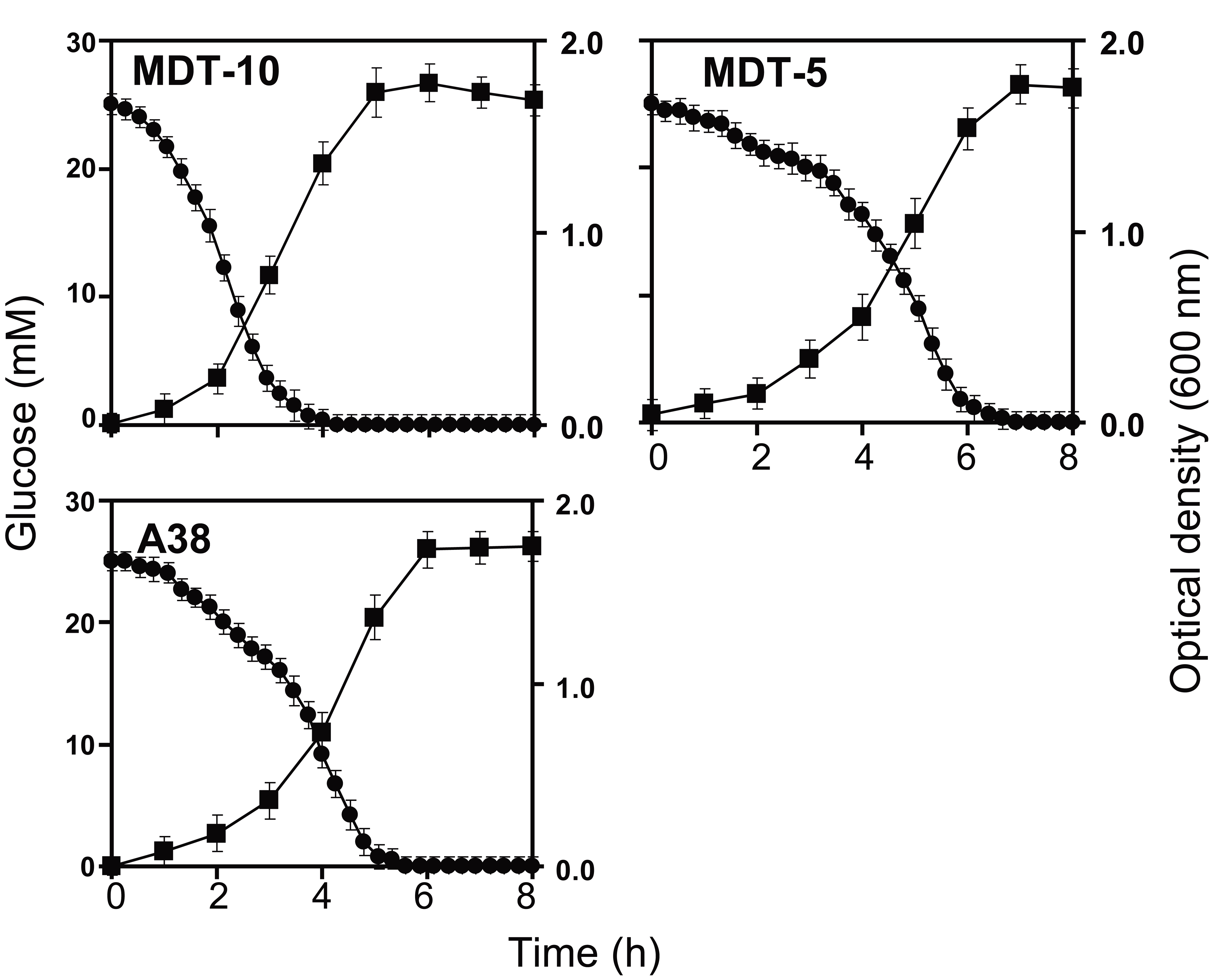

Supplement: Figure S1 — Glucose concentration and growth rate of three strains of B. fibrisolvens incubated in NMR tubes. Glucose concentration (circles) was calculated from the signal intensities observed every 5 minutes. OD (squares) was determined hourly. Mean values of triplicate experiments are shown. (0.42 MB TIF) [file pone.0004893.s001.tif]

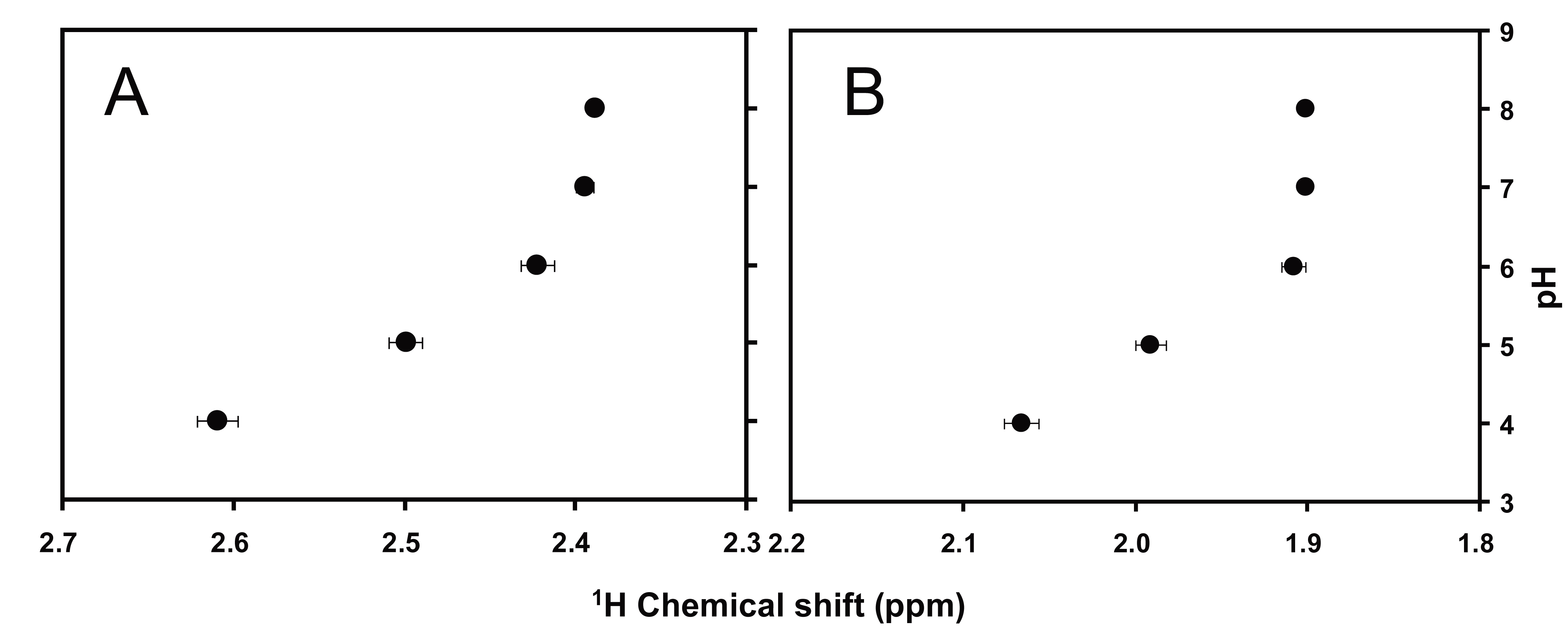

Supplement: Figure S2 — Dependence of pH on chemical shift mobility of acetate (A) and succinate (B). Mean values of triplicate experiments are shown. (0.16 MB TIF) [file pone.0004893.s002.tif]

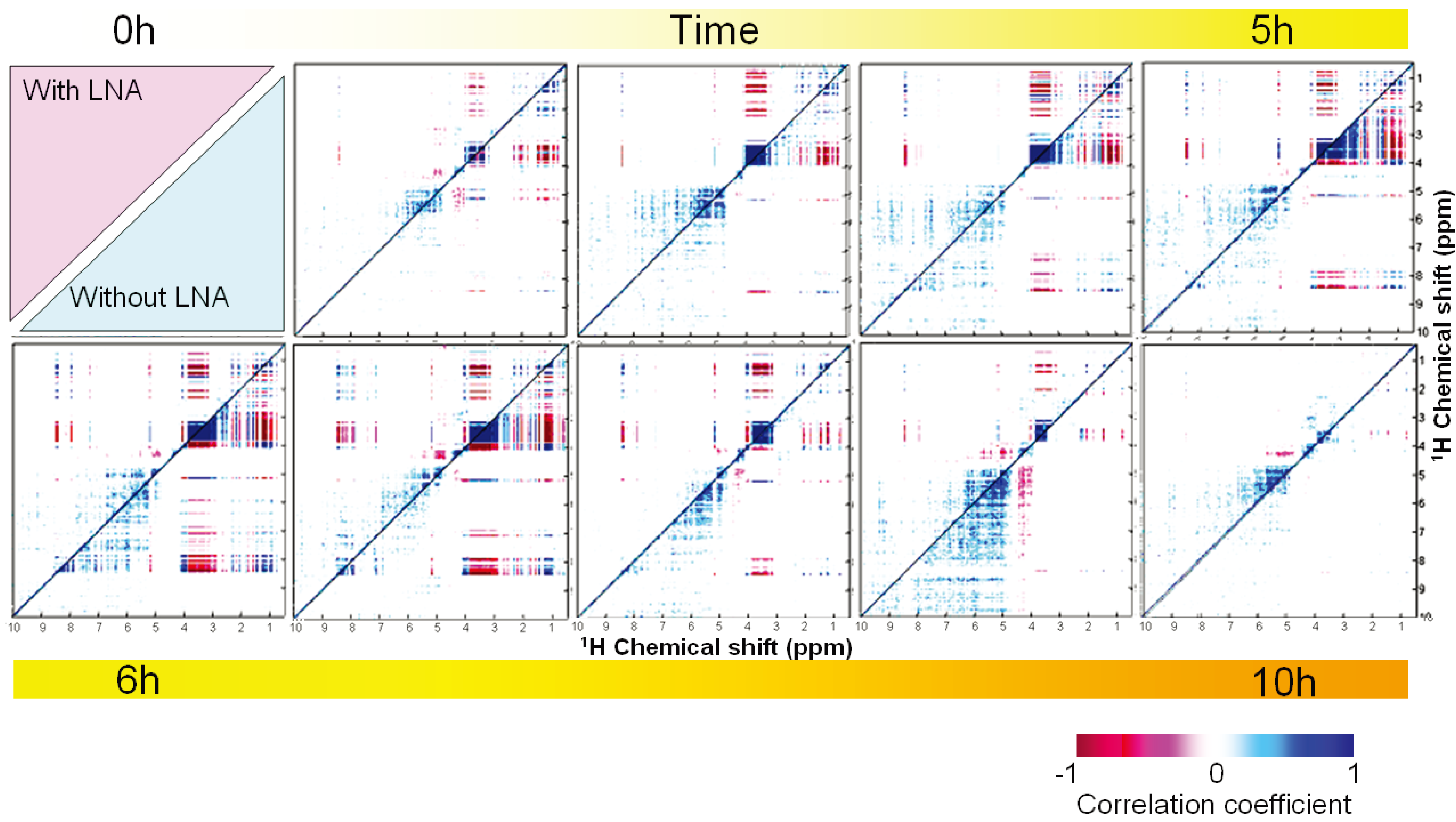

Supplement: Figure S3 — Time series of STOCSY analysis of B. fibrisolvens MDT-10 incubated with or without LNA. During these one-hour experiments, a total of twelve continuously acquired in vivo 1H-NMR spectra were used for STOCSY analysis. Contour levels (STOCSY correlations) defined by different colors are indicated at the bottom. Blue represents high positive correlation and red represents low. Data are representative of three independent experiments. (3.06 MB TIF) [file pone.0004893.s003.tif]

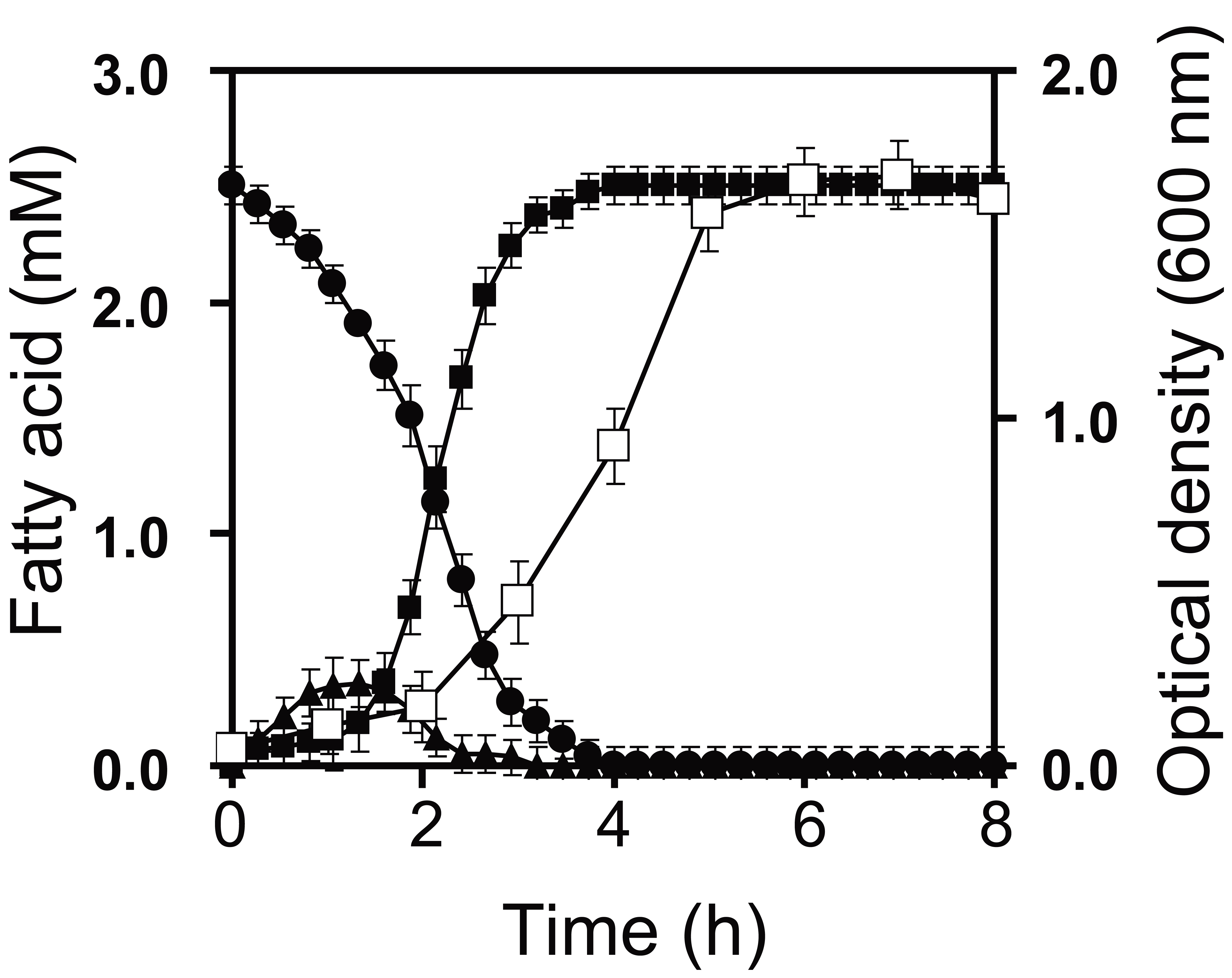

Supplement: Figure S4 — LA metabolic dynamics of B. fibrisolvens MDT-10 analyzed by time-dependent 2D-13C-HSQC RT-MT. B. fibrisolvens MDT-10 was anaerobically inoculated in an NMR tube and U-13C18 LA (2.5 mM) was added to monitor LA hydrogenation by in vivo 2D-13C-HSQC. Mean values of triplicate experiments are shown. LA (circles), CLA (triangles), VA (squares), and bacterial growth (open squares) are shown. (0.39 MB TIF) [file pone.0004893.s004.tif]

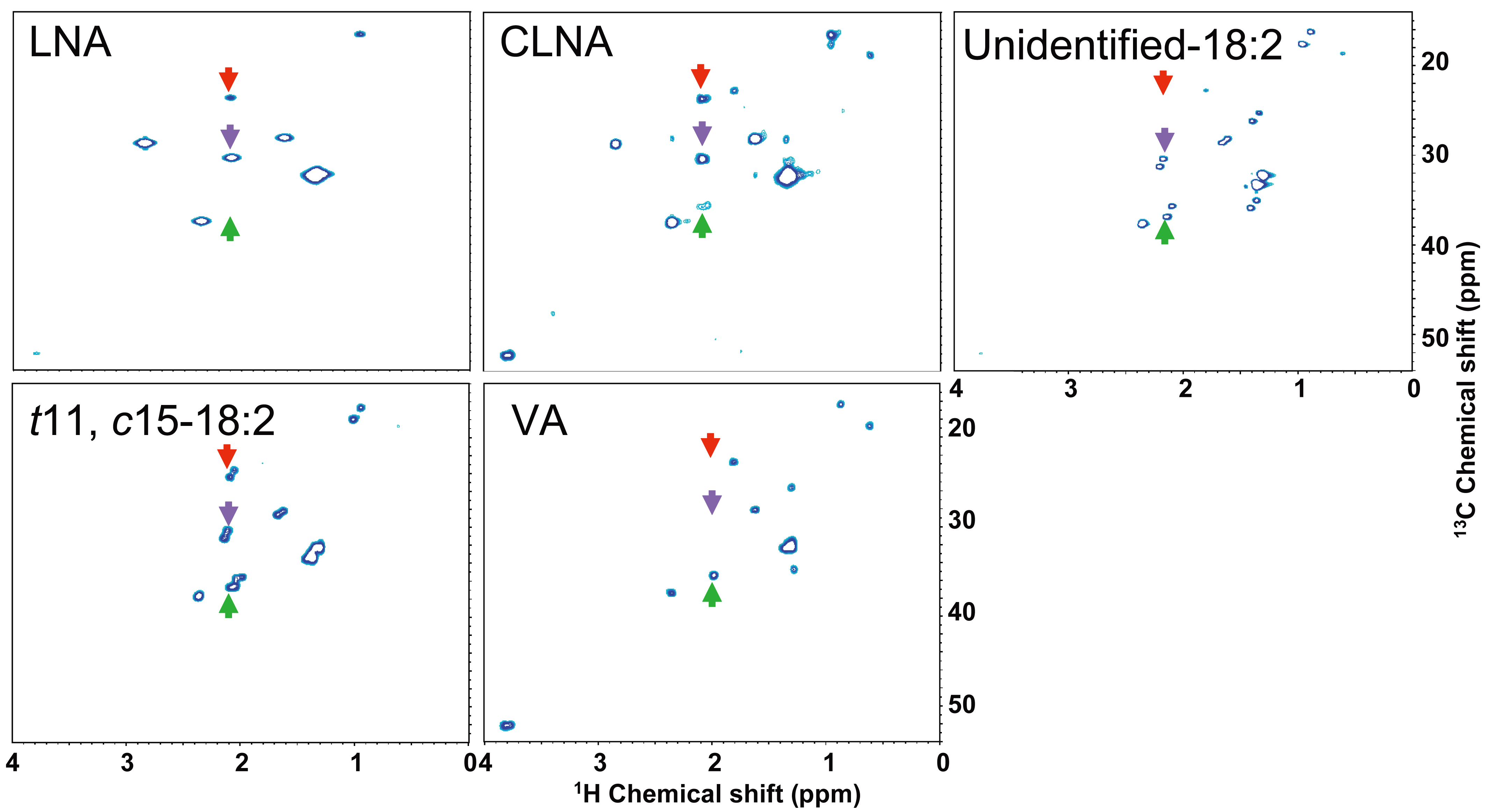

Supplement: Figure S5 — Comparison of 2D-13C-HSQC spectra obtained from LNA and its metabolites. Distinct signals are shown by arrows indicating Δ15 double bond (red), cis double bond (purple), and trans double bond (green). Unidentified 18:2 has trans and cis double bonds, but no Δ15 double bond. (0.37 MB TIF) [file pone.0004893.s005.tif]

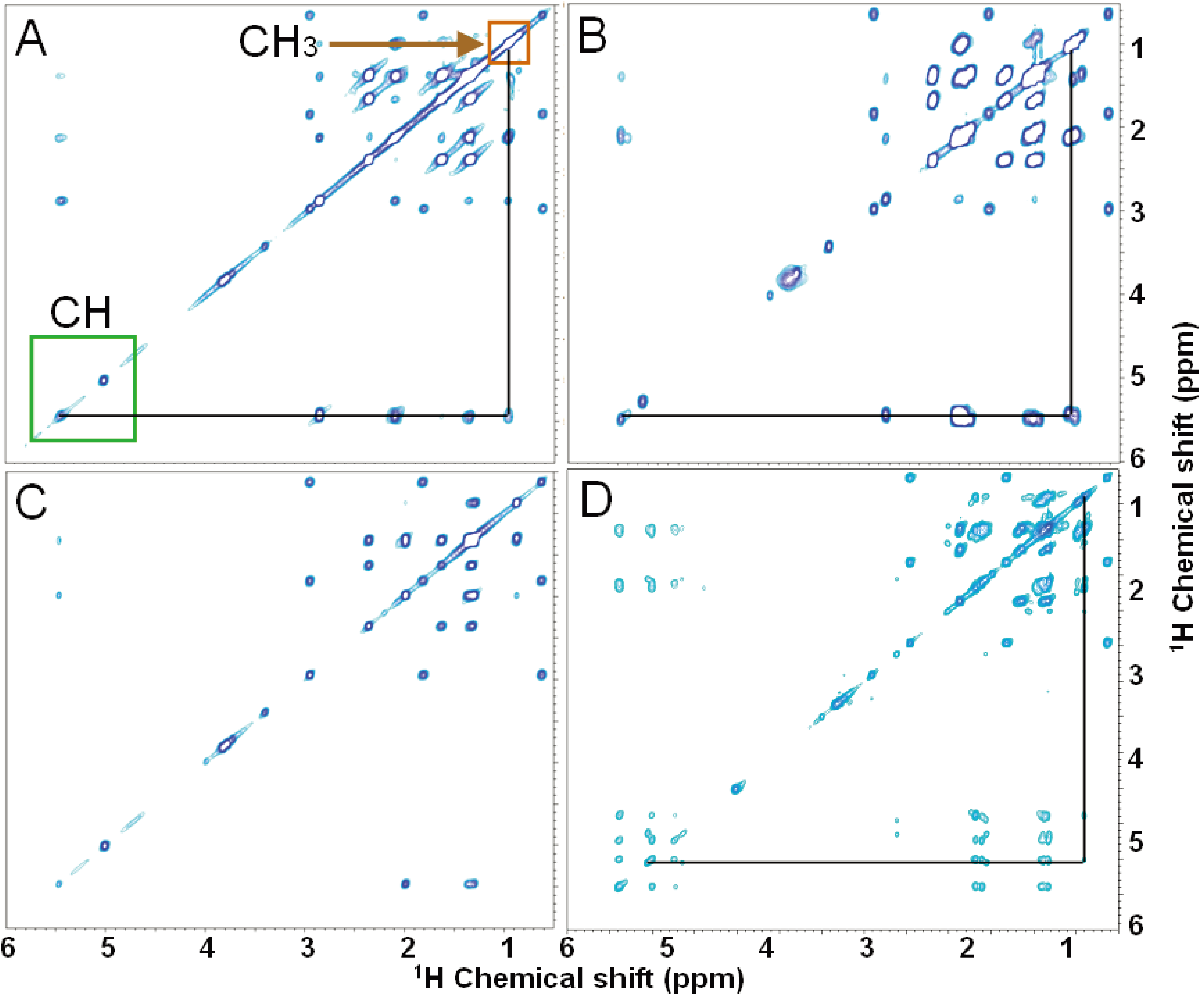

Supplement: Figure S6 — Comparison of TOCSY spectra of LNA metabolites. A: CLNA (c9, t11,c15–18:3), B: t11,c15–18:2, C: VA (t11–18:2), D: Unidentified 18:2. Black line indicates correlation between CH3 (brown square) and CH (green square). It was shown that unidentified 18:2 exhibits correlation between CH3 and CH, meaning that one of the double bonds of this FA is located more closely to the methyl group side than Δ11. (1.37 MB TIF) [file pone.0004893.s006.tif]
