# Supplementary material for: Evaluation and Characterization of Bacterial Metabolic Dynamics with a Novel Profiling Technique, Real-Time Metabolotyping
Source: PLoS One. 2009 Mar 16;4(3):e4893. doi: 10.1371/journal.pone.0004893 (PMC2654759; doi:10.1371/journal.pone.0004893)
Supplement: Table S1 — Fluctuation of signal intensities of methylene group of U-13C18 LA under various in vivo NMR conditions. (0.02 MB DOC) [file pone.0004893.s007.doc]

Table S1. Fluctuation of signal intensities of methylene group of U-^13^C_18_ LA under various *in vivo* NMR conditions.

Normalized value^1^

O.D. π/2 π/3 π/4

0.1 0.26±0.015 0.25±0.010 0.23±0.011

0.5 0.25±0.015 0.24±0.013 0.25±0.012

1.0 0.24±0.012 0.27±0.012 0.26±0.015

Vertical column shows different O.D. values of *B. fibrisolvens* in NMR tubes. Horizontal column shows different widow function values for FT processing. From up to down, π/2, π/3, π/4 shifted for sine-squared window functions. Mean values of triplicate experiments are shown.

^1^ Normalized values are shown as the relative value to internal standard.
