# Supplementary material for: Evaluation and Characterization of Bacterial Metabolic Dynamics with a Novel Profiling Technique, Real-Time Metabolotyping
Source: PLoS One. 2009 Mar 16;4(3):e4893. doi: 10.1371/journal.pone.0004893 (PMC2654759; doi:10.1371/journal.pone.0004893)
Supplement: Table S2 — Fluctuation of signal intensities of methylene group of U-13C18 LA under various pH conditions. (0.02 MB DOC) [file pone.0004893.s008.doc]

Table S2. Fluctuation of signal intensities of methylene group of U-^13^C_18_ LA under various pH conditions.

pH Normalized value^1^

4.0 0.24±0.013

5.0 0.26±0.015

6.0 0.25±0.012

7.0 0.26±0.015

^1^ Normalized values are shown as the relative value to internal standard.
